# Chemotherapy Potentially Facilitates the Occurrence of Radiation Encephalopathy in Patients With Nasopharyngeal Carcinoma Following Radiotherapy: A Multiparametric Magnetic Resonance Imaging Study

**DOI:** 10.3389/fonc.2019.00567

**Published:** 2019-07-03

**Authors:** Youming Zhang, Xiaoping Yi, Jianming Gao, Li Li, Lizhi Liu, Ting Qiu, Jinlei Zhang, Yuanchao Zhang, Weihua Liao

**Affiliations:** ^1^Department of Radiology, Xiangya Hospital, Central South University, Changsha, China; ^2^State Key Laboratory of Oncology in South China, Department of Radiation Oncology, Collaborative Innovation Center for Cancer Medicine, Sun Yat-Sen University Cancer Center, Guangzhou, China; ^3^State Key Laboratory of Oncology in South China, Collaborative Innovation Center for Cancer Medicine, Imaging Diagnosis and Interventional Center, Sun Yat-Sen University Cancer Center, Guangzhou, China; ^4^Key Laboratory for NeuroInformation of Ministry of Education, School of Life Sciences and Technology, University of Electronic Science and Technology of China, Chengdu, China; ^5^National Clinical Research Center for Geriatric Disorders, Xiangya Hospital, Central South University, Changsha, China

**Keywords:** nasopharyngeal carcinoma, radiotherapy, consolidation chemotherapy, cerebral cortex, neuroimaging

## Abstract

Radiation encephalopathy (RE) is deemed to be a disease induced only by radiotherapy (RT), with the effects of chemotherapeutic agents on the brains of nasopharyngeal carcinoma (NPC) patients being largely overlooked. In this study, we investigated structural and functional brain alterations in NPC patients following RT with or without chemotherapy. Fifty-six pre-RT, 37 post-RT, and 108 post-CCRT (concomitant chemo-radiotherapy) NPC patients were enrolled in this study. A surface-based local gyrification index (LGI) was obtained from high resolution MRI and was used to evaluate between-group differences in cortical folding. Seed-based functional connectivity (FC) analysis of resting-state fMRI data was also conducted to investigate the functional significance of the cortical folding alterations. Compared with the Pre-RT group, patients in the Post-CCRT group showed LGI reductions in widespread brain regions including the bilateral temporal lobes, insula, frontal lobes, and parietal lobes. Compared with the Post-RT group, patients in the Post-CCRT group showed LGI reductions in the right insula, which extended to the adjacent frontal lobe. Seed-based FC analysis showed that patients in the Post-CCRT group had lower FC between the insula and the left middle frontal gyrus than patients in the Pre-RT group. The follow-up results showed that patients in the Post-CCRT group had a much higher RE incidence rate (20.4%) than patients in the Post-RT group (2.7%; *P* = 0.01). These findings indicate that chemotherapy potentially facilitated the occurrence of RE in NPC patients who underwent radiotherapy.

## Introduction

Nasopharyngeal carcinoma (NPC) is a malignancy arising from the epithelium of the nasopharynx and is prevalent in southern China (1–3). Radiotherapy (RT) with or without chemotherapy is the primary treatment for patients with NPC (4); however, despite considerable improvements in RT techniques and chemotherapy regimens for patients with NPC, treatment-related complications are still not uncommon in routine clinical practice. Radiation encephalopathy (RE) is one of the most severe complications in patients with NPC who have undergone RT, and has been a subject of attention for several decades. Clinically, RE has a high incidence rate of 7–11%, and is commonly associated with severe cognitive difficulties, psychological problems, and oppressive symptoms (5, 6), which can all seriously affect patients' survival and quality of life. Given the irreversibility of RE at the time of clinical diagnosis, the early detection of treatment-related structural and functional brain alterations prior to RE is of great importance for its early identification and timely prevention.

Modern *in vivo* non-invasive imaging modalities such as magnetic resonance imaging (MRI) enable us to detect functional and structural alterations of the brain in many neuropsychiatric disorders, and a number of studies using MRI have reported treatment-induced alterations in brain structure and function in patients with NPC prior to their development of RE. For example, some functional MRI (fMRI) studies have reported that post-RT NPC patients showed altered local brain activities and intra- and/or inter-network functional connectivity (FC) in the cerebellum and cerebrum in comparison with pre-RT patients (7–10). Using voxel-based morphometry (VBM) and surface-based morphometry (SBM), several structural MRI (sMRI) studies have shown decreased gray matter (GM) volume, cortical thinning, and increased cortical surface area in specific brain regions, including the bilateral temporal lobes (6, 11–14). Using tract-based spatial statistics (TBSS), a diffusion tensor imaging (DTI) study reported impaired white matter (WM) microstructural integrity in early phase post-RT patients (15). However, these studies mainly focused on the detection of radiation-induced cerebral injury, and largely overlooked the effect of chemotherapeutic agents on brain structure and function in NPC patients. Indeed, neuropsychological studies have provided compelling evidence that systemic chemotherapy alone can induce long-term changes in cognitive function (16, 17). Furthermore, a number of MRI studies have reported chemotherapy-associated micro- and macroscopic cerebral alterations in a variety of disease populations. Specifically, by making comparisons with healthy controls, recent fMRI studies on patients with breast cancer and long-term survivors of acute lymphoblastic leukemia found chemotherapy-induced abnormal brain fMRI activity in several brain regions, including the frontal lobes (18–20). Moreover, associations between chemotherapy-induced GM atrophy, disrupted WM microstructural integrity, and clinical parameters such as the accumulated time since treatment were also observed in a number of sMRI studies of these two diseases (21–23). In patients with locally advanced NPC, the addition of chemotherapy to the treatment regimen is quite common (24). However, although it has positive roles in disease control and the eradication of subclinical micrometastasis, chemotherapy is associated with serious adverse effects on the central nervous system (25). Therefore, it is of particular interest to examine the extent of chemotherapy-related brain injury, and to determine whether chemotherapy is involved in facilitating the process of RE in patients with NPC.

In the present study, we used structural and functional MRI data to characterize GM alterations in a cohort of patients with NPC who had undergone RT (post-RT) and concomitant chemo-radiotherapy (post-CCRT), and comparing them with pre-RT NPC patients. Specifically, we first conducted surface-based local gyrification index (LGI) analysis (a morphometric characteristic thought to be both genetically and phenotypically independent of cortical thickness, and not previously explored in this disease) to compare the cortical folding patterns between the groups. Then, by choosing a cortical region showing a significant between-group difference in the LGI analysis as a region of interest (ROI), we performed seed-based FC analysis to examine the functional significance of the LGI changes. Given that chemotherapy could possibly potentiate RT of nasopharyngeal lesions by enhancing their radiosensitivity, we hypothesized that: (1) patients with NPC in the post-CCRT group would exhibit a larger range of abnormal cortical regions than those in the post-RT group; and (2) chemotherapy might facilitate the occurrence of RE.

## Materials and Methods

### Subjects

**Figure 1:**
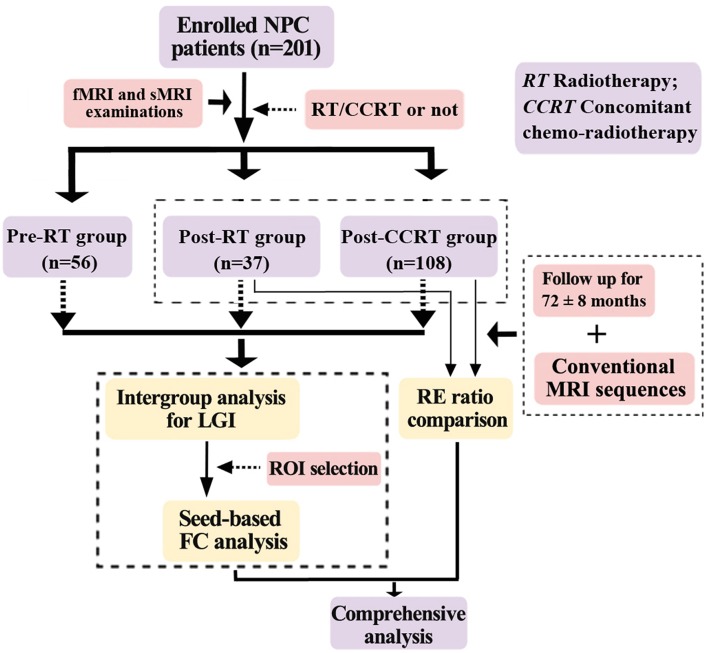
Flow diagram for the NPC patient selection, grouping, and analysis. NPC, nasopharyngeal carcinoma; RT, radiotherapy; CCRT, concomitant chemo-radiotherapy; LGI, local gyrification index; RE, radiation encephalopathy; ROI, region of interest; FC, functional connectivity.

Two hundred and one patients with pathologically confirmed NPC were enrolled in this prospective cross-sectional study. The specific procedures for this study were as follows. First, to investigate the effects of chemotherapy on brain structure and function, other possible confounding factors (such as age, sex, time intervals between RT and fMRI and sMRI examinations, RT technology, and maximum RT dosage to the temporal lobes) were minimized by ensuring that they were distributed evenly among groups. Second, each patient underwent a single fMRI and sMRI scan. Third, LGI analysis and seed-based FC analysis were performed on the fMRI and sMRI data. Fourth, using routine conventional MRI sequences, we followed up the NPC patients in the post-RT and post-CCRT groups after their fMRI and sMRI examinations for a period of 72 ± 8 months, to identify those patients who suffered from RE. Fifth, to demonstrate that chemotherapy potentially facilitated the process of RE, we performed a comprehensive analysis on the patients in the post-RT group by integrating the results of the multiparametric MRI data analysis with the presence or absence of RE during follow-up. The procedures for selection and grouping of the NPC patients are illustrated in Figure 1. The inclusion criteria for all patients in this study were as follows: (1) Pathologically confirmed NPC patients; (2) normal-appearing brain parenchyma on MRI; (3) right-handedness; (4) more than 6 years of education; and (5) an age range from 20 to 60 years. Exclusion criteria included patients with brain parenchymal invasion, brain tumors, prior substantial head trauma or surgery, neurological or psychiatric diseases, alcoholism or drug abuse, or any other major intracranial disease (6). The diagnostic criteria for RE included the following three aspects: a history of NPC with RT, typical MRI findings in the temporal lobes, and exclusion of other intracranial disease (Figure S1). Specifically, the typical MRI findings were: (1) Contrast-enhanced lesions (including nodular enhanced lesions with or without central necrosis, irregular edge enhanced lesions, and dotted and patchy enhanced lesions) on contrast-enhanced T1-weighted images. (2) White matter lesions with a high signal intensity on T2-weighted images and low signal intensity on T1-weighted images. (3) Gray matter lesions with hyperintensity in the cortex on T2-weighted images. (4) Cysts showing as round or oval well-defined thin-walled lesions of very high signal intensity on T2-weighted images. (5) Hemosiderin deposition with nodular or annular hypointense lesions on both T1- and T2-weighted images. In the assessment of follow-up MRI examinations by head and neck radiologists, patients with clinical or laboratory evidence of brain tumor, infarction, abscess, or intracranial invasion from NPCs were excluded from this study (5, 26, 27). This study was approved by the medical research ethics committee of our hospital, and written informed consent was obtained from all subjects.

For each patient, clinical data including the Karnofsky Performance Status (KPS) score, the main side of NPC, clinical stage, RT techniques, maximum dosage of RT to the temporal lobes, time intervals between RT and MRI examination, and any follow-up incidence of RE were collected, together with detailed information on the chemotherapy agents. The clinical stages of the tumors were assigned according to the 7th edition of the UICC/AJCC (2009) TNM classification (T = Tumor, N = Nodes, and M = Metastasis) (28). Intensity-modulated radiation therapy (IMRT) or conventional two-dimensional radiotherapy (2D-CRT) was performed in all the patients treated with RT, and detailed information on this is presented in Table 1. For patients in locally advanced stage (IIb to IVa–b), CCRT was recommended, either with or without neoadjuvant/adjuvant chemotherapy. Specifically, patients undergoing CCRT received cisplatin (100 mg/m^2^ intravenously on day 1). For those patients who received additional neoadjuvant chemotherapy, the chemotherapeutic agents were administrated as follows for 2 cycles: cisplatin (80 mg/m^2^ intravenously on day 1), and fluorouracil (4 g/m^2^ in continuous intravenous infusion over 120 h). For those patients who received additional adjuvant chemotherapy, the chemotherapeutic agents were administrated after RT or CCRT as follows for 3 cycles: cisplatin (80 mg/m^2^ intravenously on day 1), and fluorouracil (4 g/m^2^ in continuous intravenous infusion over 120 h).

**Table 1:** Clinical parameters.

**Items**	**Pre-RT group (*n* = 56)**	**Post-RT group (*n* = 37)**	**Post-CCRT group (*n* = 108)**	***P-*value**
**Sex**, ***n***				
Male	43 (21.4)	29 (14.4)	74 (36.8)	0.59
Female	13 (6.5)	8 (4.0)	19 (9.5)	
**Age (years)**, **mean** **±** **SD**	47.0 ± 9.0	48.5 ± 10.1	45.5 ± 7.8	0.17
**KPS score** **(median** **±** **IQR, range)**	90 ± 0, 80–90	90 ± 0, 80–90	90 ± 0, 80–90	0.59
**Main side of NPC**				
Left, *n*	17 (8.4)	8 (4.0)	20 (10.0)	0.51
Right, *n*	22 (10.9)	18 (9.0)	50 (24.9)	
Bilateral, *n*	17 (8.4)	11 (5.5)	38 (18.9)	
**Clinical staging** **(****28****)**				
I/II, *n*	14 (7.0)	29 (14.4)	23 (11.4)	<0.01^*^
III/IV, *n*	42 (20.9)	8 (4.0)	85 (42.3)	
**RT technology**				
IMRT, *n*	NA	28 (19.3)	86 (59.3)	0.61
2D-CRT, *n*	NA	9 (6.2)	22 (15.2)	
**Maximum dosage of RT for temporal lobes (Gy)**				
Left	NA	62.6 ± 7.8^a^	66.0 ± 10.3^b^	0.14
Right	NA	64.6 ± 7.0^a^	67.5 ± 8.1^b^	0.13
**Time intervals between** **RT and fMRI and sMRI** **examinations (month)**	NA	20.9 ± 25.4	16.9 ± 23.8	0.39
**RE in the follow-up**, ***n***	NA	1/37 (2.7)	22/108 (20.4)	0.01^*^

*SD, standard deviation; KPS, Karnofsky Performance Status; IQR, interquartile range; UICC, International Union against Cancer; AJCC, American Joint Committee on Cancer; RT, radiation therapy; IMRT, intensity-modulated radiation therapy; 2D-CRT, conventional two-dimensional radiotherapy; fMRI, functional MRI; sMRI, structural MRI; RE, radiation encephalopathy. (1) Data in parentheses are percentages; (2) The superscripts a and b denote data loss in 5 and 9 subjects respectively; (3) The demographic characteristics of the subjects are presented as the mean and SD with a normal distribution, median, and IQR with a non-normal distribution, or distribution of the frequency with qualitative data*.

* *P < 0.05*.

### MRI Acquisition and Image Assessment

All MRI data were obtained on a Siemens Magnetom Tim Trio 3.0-T MR scanner with a 32 channel head coil. Routine imaging studies including axial T1-weighted images, T2-weighted images, and T2-weighted fluid attenuated inversion recovery images were obtained for each subject to detect any clinically silent lesions. For each patient, the scanning sessions for the study-specific data analysis included: (1) high-resolution structural images of the whole brain using a T1-weighted 3D magnetization-prepared rapid acquisition gradient-echo sequence with 176 sagittal slices, matrix size = 256 × 256, thickness/gap = 1.0/0 mm, field of view (FOV) = 256 × 256 mm, echo time (TE) = 2.98 ms, repetition time (TR) = 2,300 ms, flip angle = 9°, and voxel size = 1.0 × 1.0 × 1.0 mm; (2) echo-planar imaging for blood oxygenation level dependent signal with 40 axial slices, thickness/gap = 3.0/0.7 mm, matrix size = 64 × 64, TR = 2,400 ms, TE = 30 ms, flip angle = 90°, and FOV = 230 × 230 mm. During scanning, subjects were instructed to remain as calm as possible and keep their eyes closed, but not to fall asleep.

### Data Pre-processing

Each structural scan was processed using the FreeSurfer package (which is freely available to the research community; http://freesurfer.net/) to obtain the LGI. Briefly, the LGI map was obtained in four steps. First, the pial surface was reconstructed in 3-dimensional space. Second, the outer surface was obtained from the outer hull, which tightly wraps the pial surface. Third, the LGI was calculated for each vertex on the outer surface as the inverse of the ratio of the area of the circular region centered on this vertex and the area of the corresponding region of the pial surface. Thus, the LGI can be used to quantify the cortical surface invaginated in the sulci and measure the spatial frequency of the cortical gyrification and depth of the sulci. Fourth, the LGI map was obtained by propagating the LGI values from the outer surface to the pial surface. For comparison purposes, all of the individual reconstructed cortical surfaces were aligned to an average template (fsaverage) using a surface-based registration algorithm. The LGI maps were then resampled and smoothed with a heat kernel of 10-mm width.

### Functional Connectivity Analysis

All resting-state fMRI data were preprocessed using DPARSFA (Data Processing Assistant for Resting-State fMRI Advanced Edition) (29). For each subject, the first 10 volumes of each scan were discarded to allow for magnetization equilibrium. The subsequent preprocessing then included slice timing, head motion correction, spatial normalization to the Montreal Neurological Institute (MNI) template, resampling to 3 × 3 × 3 mm, smoothing with a 4-mm Gaussian kernel to decrease spatial noise, temporal band-pass filtering, and regressing out of nuisance signals including head motion parameters, WM, and cerebrospinal fluid.

To examine the functional significance of the LGI alterations, a cortical region showing significant differences between the post-RT and post-CCRT groups in the LGI analysis was extracted as a seed region for further seed-based functional connectivity analysis. Specifically, we extracted the mean time series of the seed regions for each subject, and then correlated this mean time series with those of all the other voxels throughout the whole brain. The resulting correlation images were then converted to z-score images using Fisher's r-to-z transformation to improve normality.

### Statistical Analyses

Group differences in age were assessed using one-way analysis of variance, and gender, main side of the nasopharyngeal tumor, radiation therapy technology, and RE ratios in the follow-up were analyzed with the Chi-squared test. The KPS scores were compared between the patient and control groups using the Kruskal–Wallis non-parametric test.

Vertex-by-vertex contrasts of the LGI maps were performed for each pair of groups using the SurfStat package (http://www.math.mcgill.ca/keith/surfstat/). Specifically, each contrast was entered into a vertex-by-vertex general linear model (GLM) with gender and age as covariates. A threshold of *P* < 0.01 was then used to define clusters demonstrating a difference. Subsequently, a cluster-wise-corrected *P*-value was obtained for each cluster using random field theory (RFT). The significance level of the clusters was set at *P* < 0.05 after correction for multiple comparisons.

In the post-treatment group (including post-RT and post-CCRT groups), vertex-wise partial correlation analyses were performed to determine the relationships between LGI and the clinical data such as the ipsilateral maximum dosage of radiation-treatment (MDRT) to the temporal lobe, while adjusting for gender and age. Then, the procedures performed for the between-group analysis on LGI were repeated, to correct for multiple comparisons and to report the results of the correlation analysis.

In the FC analysis, the GLMs used in the between-group LGI analysis were adopted to test for FC differences between the three groups. A threshold of *P* < 0.005 was used to define clusters showing significant differences. Subsequently, a cluster-wise-corrected *P*-value was obtained for each cluster using Gaussian random field theory. The significance level for clusters was set at *P* < 0.05 after correction for multiple comparisons. In the post-treatment group (including post-RT and post-CCRT groups), partial correlation analyses were performed between the clinical data such as MDRT, and the average z-scores of regions with significant FC differences, while adjusting for gender and age.

## Results

### Clinical Data Analyses

The clinical data are presented in Table 1. The clinical parameters of sex, age, KPS score, main side of NPC, RT technology, and time interval between RT and MRI examination were not significantly different between the pre-RT, post-RT, and post-CCRT groups (*P* = 0.59, 0.17, 0.59, 0.51, 0.61, and 0.39, respectively). In the patients treated by RT, the MDRT to the temporal lobes was also distributed evenly between the post-RT and post-CCRT groups (*P* = 0.14 for the left temporal lobe, 0.13 for the right temporal lobe). However, there were significant differences in clinical staging between the pre-RT, post-RT, and post-CCRT groups (*P* < 0.01).

During the follow up, 23 patients in the post-treatment group including post-RT and post-CCRT groups suffered from RE, giving an incidence rate of 15.9% (23/145). In the post-RT group, only one patient was diagnosed with RE, giving an incidence rate of 2.7% (1/37). In the post-CCRT group, 22 patients were diagnosed with RE, giving an incidence rate of 20.4% (22/108). The RE incidence rate was significantly higher in the post-CCRT group than in the post-RT group (*P* = 0.01). The mean time interval between the end of RT and the time at which RE was confirmed was 42.3 ± 12.2 months.

### Intergroup LGI Analysis

**Figure 2:**
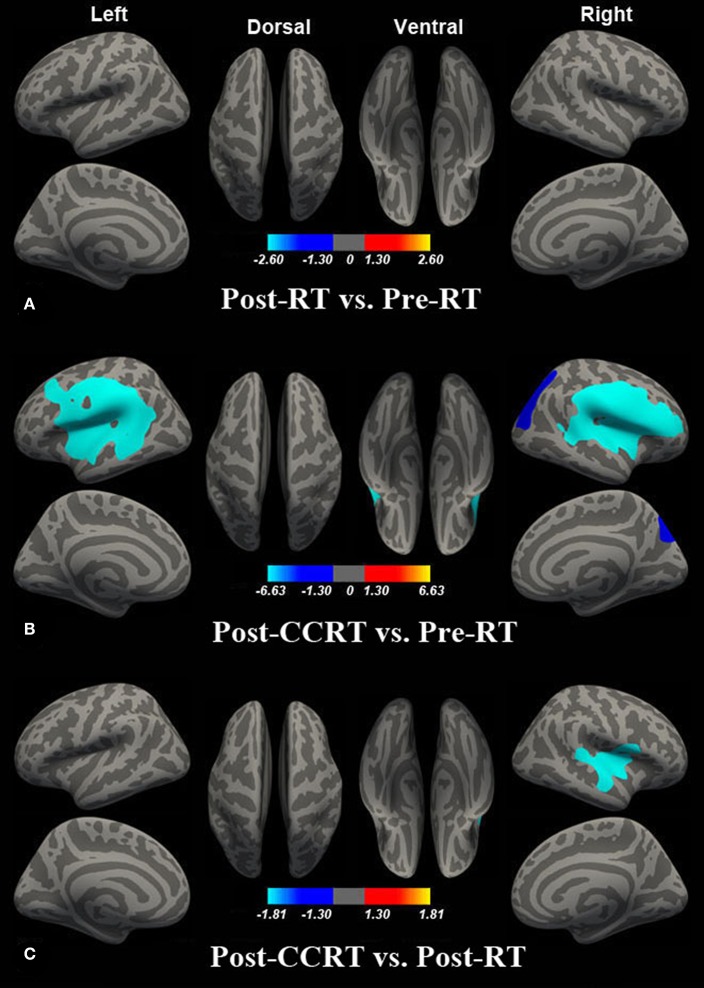
Between-group differences in LGI. Compared with the pre-RT group, no significant LGI difference was observed in patients in the post-RT group **(A)**. Compared with the pre-RT group, patients in the post-CCRT group showed widespread LGI reductions in the right inferior and superior parietal lobule, bilateral superior temporal gyrus (STG), left middle temporal gyrus (MTG), bilateral insula, and precentral, postcentral, supramarginal, inferior, and middle frontal gyri **(B)**. Compared with the post-RT group, patients in the post-CCRT group showed significant LGI reduction in the right insula, extending to the adjacent frontal lobe and superior temporal gyrus **(C)**. Colored areas denote regions where a significant difference in LGI was observed between the indicated groups. The absolute value or the magnitude of the color bar is corresponding to log10 (1/P), and the sign of the color bar denotes the directions of the changes.

Compared with the pre-RT group, patients in the post-CCRT group showed widespread LGI reductions in the right inferior and superior parietal lobule, bilateral superior temporal gyrus (STG), left middle temporal gyrus (MTG), bilateral insula, and precentral, postcentral, supramarginal, inferior, and middle frontal gyri (Figure 2B; Table S1). Compared with the post-RT group, patients in the post-CCRT group showed significant LGI reductions in the right insula, with these extending to the adjacent frontal lobe and superior temporal gyrus (Figure 2C; Table S2). No significant LGI differences were observed between the post-RT and pre-RT groups (Figure 2A). In the post-treatment patient groups (including post-RT and post-CCRT groups), no significant correlations were observed between the LGI and clinical data, including the time interval between RT and MRI examination and the MDRT to the temporal lobes.

### Functional Connectivity Analysis

**Figure 3:**
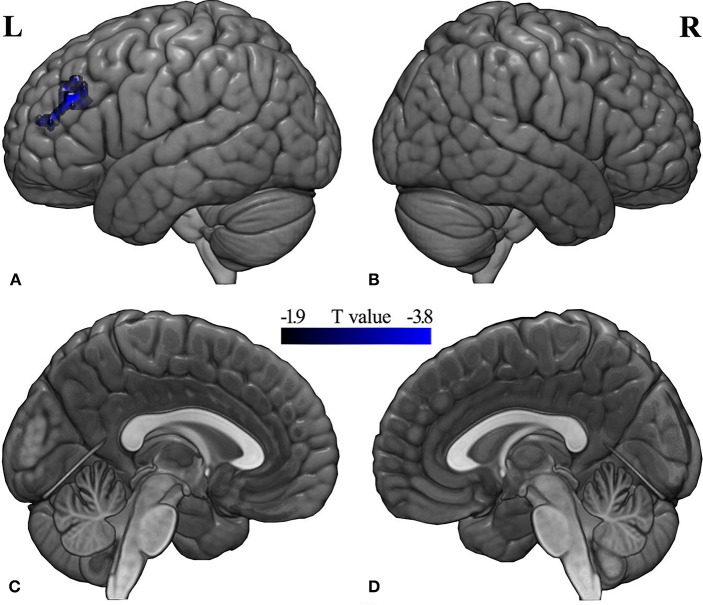
Between-group differences in seed-based FC analysis. Compared with the pre-RT group, patients in the post-CCRT group showed significantly decreased functional connectivity between the left middle frontal gyrus and the brain region of interest from the LGI analysis [**(A)** left lateral; **(B)** right lateral; **(C)** left medial; **(D)** right medial]. A *t*-score color bar is shown at the bottom. L, left; R, right.

Compared with the pre-RT group, patients in the post-CCRT group showed significantly decreased functional connectivity between the left middle frontal gyrus (MFG) and the region of interest selected according to the LGI analysis (Figure 3; Table S3). No significant intergroup FC difference was observed for the other pairwise group combinations. In the post-treatment patient groups (including post-RT and post-CCRT groups), no significant correlations were observed between the MDRT to the temporal lobes and the regions with significant FC differences.

## Discussion

In the present study, we used both LGI and FC to examine the effects of chemotherapy on the normal-appearing GM of NPC patients. Compared with the pre-RT group, patients in the post-CCRT group showed significant LGI reductions in widespread brain regions including the bilateral temporal lobes, insula, and frontal and parietal lobes, while patients in the post-RT group did not, which suggests that chemotherapy may enhance RT-associated brain damage. Moreover, compared with the post-RT group, patients in the post-CCRT group showed significant LGI reductions in the right insula, which extended to the adjacent frontal lobe, indicating that chemotherapy may be responsible for such cortical alterations. Interestingly, compared with the pre-RT group, the seed-based FC analysis showed that patients in the post-CCRT group had significantly decreased FC between the left MFG and the brain region showing significant LGI differences between the post-CCRT and post-RT groups, suggesting that these brain regions might be anatomical sites underlying the functional deficits observed in radiation encephalopathy. More importantly, the follow-up results showed that patients receiving CCRT had a much higher rate of RE than patients receiving RT alone, indicating that chemotherapy potentially facilitated the occurrence of RE. These findings may contribute to a better understanding of the underlying neural mechanisms behind RE.

### LGI Reductions in the Post-CCRT vs. Pre-RT Group

In comparison with the pre-RT group, we found significantly decreased LGI in the bilateral STG/MTG in NPC patients treated with CCRT. These findings are in line with previous structural studies, which reported significant alterations in GM volume, cortical surface area, and cortical thickness in NPC patients following their treatment (6, 11, 13, 14). Some DTI studies also provided evidence of WM damage in NPC patients following treatment (7, 15, 30), as reflected by altered fractional anisotropy (FA) and/or mean diffusivity (MD) in the temporal lobes. Functional alterations have also been documented in these brain areas of NPC patients following treatment, including a magnetic resonance spectroscopy (MRS) study that demonstrated significantly lower bilateral temporal lobe NAA/Cr and NAA/Cho ratios in NPC patients within 1 year after RT (30). Using fractional amplitude of low-frequency fluctuation (fALFF), Ding et al. examined NPC patients at different stages after RT and reported altered local brain activity in several brain regions, including the bilateral temporal lobes (7). In the current study, the observed cortical folding abnormalities measured by LGI provide further structural evidence for the involvement of the temporal lobes prior to RE. Given that the temporal lobes receive the highest radiation dose and have been reported to be associated with the ipsilateral MDRT (6), it is tempting to speculate that the measured LGI reductions in these regions were mainly induced by the RT.

We also detected significant LGI reductions in the parietal lobe in post-treatment NPC patients, including in the right inferior and superior parietal lobule, and bilateral supramarginal gyrus. It is well-known that the inferior parietal lobule and supramarginal gyrus are important components of the default mode network (DMN) (31–33), and it is therefore tempting for us to speculate that functional abnormalities in the DMN occurred in NPC patients after treatment, which is consistent with the findings of previous studies. For example, compared with pre-RT NPC patients, a structural MRI study reported altered cortical thickness in the inferior parietal lobule, precuneus, and isthmus of the cingulate cortex, indicating that these anatomical structures may be the sites underlying DMN dysfunction in post-treatment NPC patients (13). A multiparametric MRI study reported a characteristic “disruption and recovery” FC pattern within the DMN, reflecting the cognitive decline, psychological disorders, and mood disorders commonly observed in NPC patients following their treatment (7). Recently, a longitudinal resting state fMRI study used independent component analysis (ICA) to document a significant FC reduction in the anterior cingulate cortex within the DMN (10). Therefore, together with the findings of the previous studies (7, 10, 13), our observation of LGI reductions in the parietal lobe may reflect the high degree of cognitive and emotional changes induced by chemotherapeutic agents and/or radiation exposure.

Our finding of significant LGI reductions in the precentral and postcentral gyrus in patients with NPC treated with CCRT are consistent with findings reported in previous studies using other techniques. Using VBM, a structural MRI study reported significantly reduced gray matter volume in the right precentral gyrus of NPC patients after they underwent RT (14). An SBM study also documented altered cortical thickness in several brain regions in the early- and late-delayed stage after RT, including in the left precentral gyrus, while a resting state fMRI study found altered FCs in the sensorimotor network in post-RT NPC patients (9, 13). Taken together, these findings of functional and morphological alterations in the precentral and postcentral gyrus indicate motor and sensory dysfunctions in NPC patients who have undergone treatment. In fact, motor and sensory deficits are common symptoms in post-RT NPC. Previous clinical studies have demonstrated that post-treatment NPC patients exhibited swallowing impairment, bulbar palsy, and pain, reflecting sensorimotor function deficits (5, 34, 35). The exact reasons why the precentral and postcentral gyri exhibited the LGI reductions are still unclear, although they might occur as a result of secondary changes after radiation-induced injuries to the brainstem. Physiologically, the primary motor and sensory pathways traverse through the brainstem, and the abnormalities in the precentral and postcentral cortex may therefore be secondary to damage to primary motor and sensory pathways caused by irradiation of the brainstem (13, 36).

### LGI Reductions in Post-CCRT vs. Post-RT Groups

Compared with the post-RT group, patients in the post-CCRT group showed significant LGI reductions, with these occurring mainly in the right insula and extending to the adjacent frontal lobe. In combination with the finding of abnormal LGI reductions in the bilateral frontal lobes obtained from the intergroup comparison between the post-CCRT and pre-RT groups, we attributed the LGI abnormalities in these brain regions to chemotherapy-related neurotoxic effects.

The altered LGI in the right insula was obtained by subtracting the effect of the RT alone from the CCRT, and it is therefore tempting to infer that such structural changes might be induced mainly by chemotherapeutic agents. However, a recent resting-state fMRI study observed irradiation dose-dependent disruption of FC in the right insula (10). Such a discrepancy may result from differences in demographic characteristics, the grouping scheme, or the clinical stage of the patient groups; however, its exact cause remains unknown, and requires further investigation. In contrast, the cerebral morphological and functional alterations in the bilateral frontal lobes have been well-documented in previous neuroimaging studies examining chemotherapy-related cerebral function or structural alterations. For example, two prospective morphological MRI studies used VBM to show significant GM volume reductions in the frontal lobes of patients with breast cancer shortly after chemotherapy (21, 22). Similarly, altered structural and functional metrics in the frontal lobes have also been observed in long-term survivors of childhood acute lymphoblastic leukemia following systemic chemotherapy (19, 37, 38). Taken together, although we cannot rule out the possibility of RT effects in this cross-sectional study, we speculate that the decreased LGI in the right insula and bilateral frontal lobes may be partially attributable to chemotherapy.

Of note, significant LGI reductions were only found in the right hemisphere in the intergroup comparison between the post-CCRT and post-RT groups. However, such a finding does not mean that the left hemisphere was not affected by chemotherapy. Indeed, as can be seen from the t map of the contrast (post-CCRT group vs. post-RT group) (Figure S2), the affected brain regions in the two hemispheres were roughly symmetrical. The seemingly asymmetrical pattern of cortical folding alterations could simply be a byproduct of the adopted thresholding strategy, i.e., some alterations can survive the correction for multiple comparisons, while others not.

### Functional Connectivity Analysis

To explore the functional significance of the gyrification abnormality, seed-based functional connectivity analysis was conducted for the three groups. Compared with the pre-RT group, patients in the post-CCRT group showed decreased FC between the left MFG and the region of interest (mainly in the right insula). This finding is partially supported by a previous multiparametric study showing altered local brain activity in the insula and MFG in post-RT NPC patients (7). Additionally, a structural MRI study demonstrated cortical thickness alterations in the insula and MFG in post-treatment NPC patients, indicating that these may be the anatomical sites underlying the functional deficits (such as impaired language abilities and multimodal information integration dysfunction) observed in this disease (13).

Functionally, the MFG is regarded as one of the secondary language areas implicated in the nuances of language expression, such as those involved in verbal working memory and verbal fluency (39), and impairments in list-generating fluency and language abilities are common clinical manifestations in NPC patients following their treatment (40, 41). The insula is a multimodal area and has been implicated in both somatosensory and affective aspects of subjective perception in a certain emotional status (42–44). Specifically, the insular cortex is involved in salience, pain intensity, and anticipation, and negative emotions such as anxiety (45). In clinical practice, various emotional and somatosensory problems including anxiety, headache, and bulbar palsy have been reported in NPC patients following treatment (5, 6, 34). Given that brain functions are shaped and constrained by the underlying brain anatomy (46), we inferred that the observed abnormal MFG-insula FC in this study may reflect injured WM fiber tracts in post-CCRT NPC patients. In fact, WM abnormalities have frequently been reported after CCRT for NPC. For example, several neuropathological studies demonstrated widespread WM degeneration such as demyelination, atrophy, and astrogliosis in the brains of humans and animals receiving chemotherapy and radiation (47–50). Moreover, using TBSS, one recent DTI study reported significantly lower FA and higher MD in the frontal WM fiber tracts of NPC patients at 6 and 12 months after RT (15). Therefore, although we cannot rule out the involvement of other theories, it is tempting to speculate that the altered MFG–insula FC may be closely related to impairment of the underlying fiber tracts (51, 52).

### Chemotherapy Potentially Facilitated the Occurrence of RE

Intriguingly, we found that patients in the post-CCRT group had a higher RE incidence rate than those in the post-RT group, as evidenced by the results of the long-term follow-up, suggesting that chemotherapy might facilitate the occurrence of RE. In fact, although it is well-documented that chemotherapy (concurrent chemoradiotherapy, neoadjuvant, and adjuvant chemotherapy) can enhance the sensitivity of RT, reduce tumor volume, and improve blood supply in tumor cells in nasopharyngeal lesions (53–55), the synergistic side effects in the brain have never been investigated. Our study provides preliminary research in this field, and if the current findings are confirmed in future prospective longitudinal studies, they will have vital clinical significance, for they would contribute to the formulation of reasonable treatment strategies and the adjustment of follow-up plans.

This study is subject to some limitations that should be addressed. First, although clinical parameters such as the RT technology and the radiation dose to the temporal lobes were consistent between groups in this study, caution must be applied when interpreting the results of the observed LGI and FC alterations, because of the cross-sectional design. However, the study can still generate the perspective that chemotherapy could facilitate the occurrence of RE, providing a new clue for future research. Second, because of the lack of a subject group receiving only chemotherapy, we cannot determine which factor (RT or chemotherapy) was responsible for the LGI or FC abnormalities. Future studies with the inclusion of NPC patients receiving chemotherapy alone are warranted to further pursue this issue. Third, a relatively small sample size in the pre-RT and post-RT groups lowered the statistical power, resulting in differences not surviving strict corrections for multiple comparisons. Future studies with larger sample sizes are needed to replicate the current findings. Fourth, the absence of detailed evaluations of the patients' psychological status, quality of life, cognitive functions, and specific symptoms, weakens the interpretability of our findings.

## Conclusion

In this study, we investigated LGI and FC changes in NPC patients receiving RT and/or chemotherapy. We found that patients in the post-CCRT group exhibited altered LGI and FCs in a wider range of brain regions than patients receiving RT alone. Furthermore, the patients in the post-CCRT group demonstrated a higher incidence rate of RE during follow-up. Collectively, our findings indicate that chemotherapy might facilitate the occurrence of RE, providing new insights into the pathogenesis of RE in post-treatment NPC patients.

## Ethics Statement

This study was carried out in accordance with the recommendations of GCP and ICH-GCP, Ethic Committee of Xiangya Hospital, Central South University with written informed consent from all subjects. All subjects gave written informed consent in accordance with the Declaration of Helsinki. The protocol was approved by the Ethic Committee of Xiangya Hospital, Central South University (NO. 201101006).

## Author Contributions

YoZ, LiL, WL, and YuZ conceived and designed the experiments. YoZ, LiL, JG, JZ, LizL, and TQ analyzed the data. YoZ, XY, and YuZ contributed materials and analysis tools. YoZ, WL, and YuZ wrote the paper. All authors read and approved the final manuscript.

### Conflict of Interest Statement

The authors declare that the research was conducted in the absence of any commercial or financial relationships that could be construed as a potential conflict of interest.
