# Supplementary material for: Chemotherapy Potentially Facilitates the Occurrence of Radiation Encephalopathy in Patients With Nasopharyngeal Carcinoma Following Radiotherapy: A Multiparametric Magnetic Resonance Imaging Study
Source: Front Oncol. 2019 Jul 3;9:567. doi: 10.3389/fonc.2019.00567 (PMC6618298; doi:10.3389/fonc.2019.00567)
Supplement: Supplementary file 1 [file Data_Sheet_1.PDF]

Supplementary tables

Table S1. Clusters showing group LGI differences between Post-CCRT and Pre-RT

Brain regions	MNI coordinates			Side	Cluster size (vertices)	Peak T values	P value
	X	Y	Z				
STG, insula, pre-/postcentral, supramarginal gyrus, IFG, MFG	50	7	12	R	29883	4.73	2.36E-07
IPG, SPL	21	-74	46	R	7672	3.79	5.44E-04
MTG, STG, insula, pre-/postcentral, supramarginal gyrus, IFG, MFG	-60	-28	37	L	31704	4.07	2.39E-07

Note: STG Superior temporal gyrus; IFG inferior frontal gyrus; MFG middle frontal gyrus; IPL Inferior parietal lobule; SPL superior parietal lobule; MTG Middle temporal gyrus; MNI Montreal Neurological Institute; L left; R right.

Table S2. Clusters showing group LGI differences between Post-CCRT and Post-RT

Brain regions	MNI coordinates			Side	Cluster size (vertices)	Peak T values	P value
	X	Y	Z				
Insula, superior temporal gyrus and precentral gyrus	56	7	6	R	8259	3.29	1.60E-02

Note: MNI Montreal Neurological Institute; R right.

Table S3. Clusters showing group FC differences between Post-CCRT and Pre-RT

Brain regions	MNI coordinates			Side	Cluster size (mm^3)	Peak T values	P value
	X	Y	Z				
Middle frontal gyrus	-36	33	27	L	2727	-3.80	< 0.05

Note: MNI Montreal Neurological Institute; L left.
