# Supplementary material for: Chemotherapy Potentially Facilitates the Occurrence of Radiation Encephalopathy in Patients With Nasopharyngeal Carcinoma Following Radiotherapy: A Multiparametric Magnetic Resonance Imaging Study
Source: Front Oncol. 2019 Jul 3;9:567. doi: 10.3389/fonc.2019.00567 (PMC6618298; doi:10.3389/fonc.2019.00567)
Supplement: Supplementary file 2 [file Data_Sheet_2.PDF]

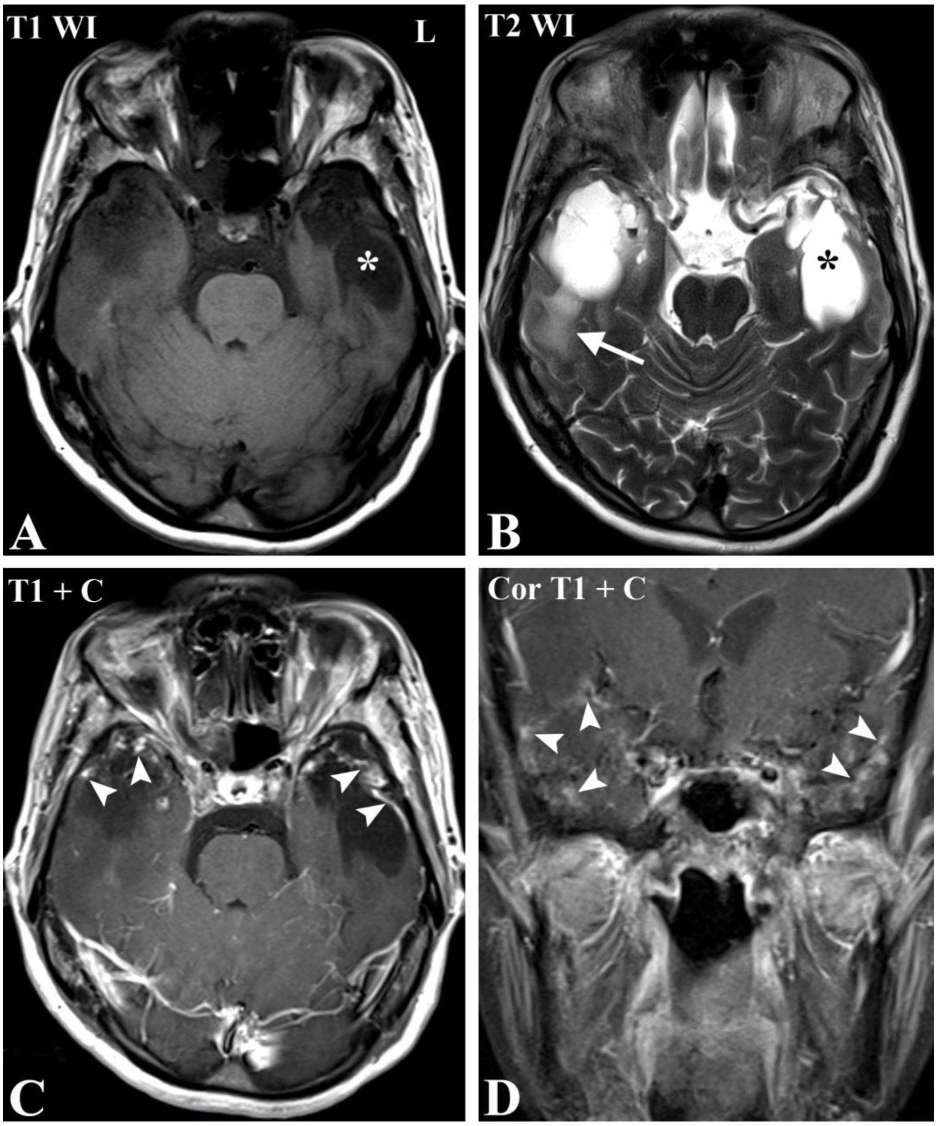

Fig. S1

Fig. S1. MRI findings of RE in a 46-year-old man with NPC after concurrent chemoradiotherapy (A, B, C, and D). Round or oval lesions of low signal intensity on T1-weighted images, very high signal intensity on T2-weighted images (A, B, asterisk) are observed in bilateral temporal lobes. Patchy edema area displays slightly high signal intensity on T2-weighted images (B, white arrow). The lesions in the bilateral temporal lobes also show irregular edge contrast enhancement after contrast agent injection (C, D, white arrowheads). Note: RE, radiation encephalopathy; NPC, nasopharyngeal carcinoma; L, left; T1 + C, T1 weighted imaging after contrast agent injection; Cor, coronal.

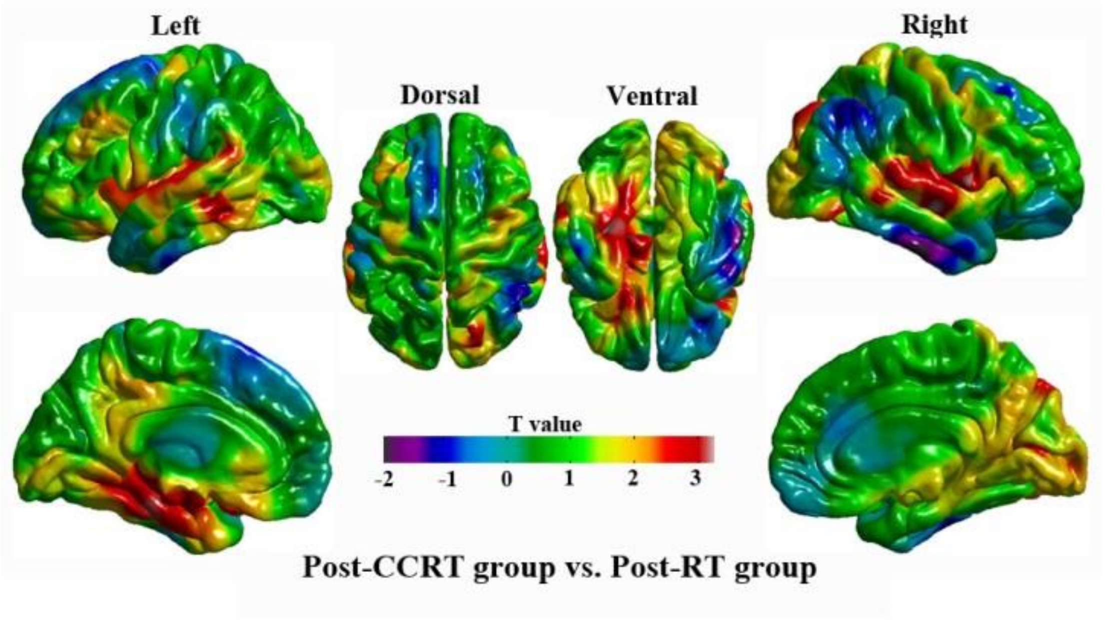

Fig. S2

Fig. S2. The t map for the intergroup comparison between the Post-CCRT and Post-RT groups. Significance is represented on a t-score scale, where positive values (warm colors) are assigned to the Post-CCRT > Post-RT and negative values (cold colors) are assigned to the Post-CCRT < Post-RT.
